# Expectation affects learning and modulates memory experience at retrieval

**DOI:** 10.1016/j.cognition.2018.07.010

**Published:** 2018-11

**Authors:** Alex Kafkas, Daniela Montaldi

**Affiliations:** Memory Research Unit, Division of Neuroscience and Experimental Psychology, School of Biological Sciences, University of Manchester, UK

**Keywords:** Context, Expectation, Familiarity, Recollection, Similarity, Pattern separation

## Abstract

Our ability to make predictions and monitor regularities has a profound impact on the way we perceive the environment, but the effect this mechanism has on memory is not well understood. In four experiments, we explored the effects on memory of the expectation status of information at encoding or at retrieval. In a rule-learning task participants learned a contingency relationship between 6 different symbols and the type of stimulus that followed each one. Either at encoding (Experiments 1a and 1b) or at retrieval (Experiments 2a and 2b), the established relationship was violated for a subset of stimuli resulting in the presentation of both expected and unexpected stimuli. The expectation status of the stimuli was found to have opposite effects on familiarity and recollection performance, the two kinds of memory that support recognition memory. At encoding (Experiments 1a and 1b), the presentation of expected stimuli selectively enhanced subsequent familiarity performance, while unexpected stimuli selectively enhanced subsequent recollection. Similarly, at retrieval (Experiments 2a and 2b), expected stimuli were more likely to be deemed familiar than unexpected stimuli, whereas unexpected stimuli were more likely to be recollected than were expected stimuli. These findings suggest that two separate memory enhancement mechanisms exist; one sensitive and modulating the accuracy of memory for the contextually distinctive or unexpected, and the other sensitive to and modulating the accuracy of memory for the expected. Therefore, the degree to which information fits with expectation has critical implications for the type of computational mechanism that will be engaged to support memory.

## 1 Introduction

A fundamental function of the human mind is the ability to infer predictions and form expectations (Bar, 2009, Hunt and Aslin, 2001, Schacter et al., 2007). Apart from monitoring regularities in the environment, our brains also need to be able to learn from, and thereby adapt to, both expected and unexpected stimulus encounters. An important outstanding question, therefore, relates to the effect the level of expectation can have on the mechanisms brought into play at encoding and retrieval, and how these may selectively enhance different kinds of memory.

Indeed, adaptive behaviour dictates that the memorability of important, motivational or salient events is achieved by triggering a repertoire of orienting behavioural outcomes and by engaging a specialised network of brain regions (Kafkas and Montaldi, 2015a, Lisman and Grace, 2005, Shohamy and Adcock, 2010). On the other hand, evidence also supports the idea that expected information (e.g., as with schemas) can have an advantage in memory (e.g., Bein et al., 2015, Craik and Tulving, 1975). Expectation embedded in a sequence of events has been shown to affect perceptual discrimination and object categorisation (e.g., Bollinger et al., 2010, Posner et al., 1980, Puri and Wojciulik, 2008). Nevertheless, the way expectation affects memory formation and retrieval has not been explored systematically. Understanding the interaction between expectation and new learning, or the retrieval of already learned information, can critically inform key areas of application, such as organised learning settings (e.g. educational institutions). In the current paper, a set of experiments is reported which systematically explored the effect of expectation on different kinds of memory using a paradigm especially designed to investigate memory formation and retrieval under different levels of expectation.

### 1.1 Context, expectation, familiarity and recollection

Here we define expectation as the “frame of reference” that describes the sequence of events within a context of temporally related events. Therefore, after establishing that an event A is always followed by event B; an event C is unexpected when following A, while B, is the expected event within the ABC context. In the following experiments, we manipulated expectations for newly learned sequences of events (contexts) and we investigated their effect on memory. The term *context* is used in different ways in the memory literature and in relation to episodic memory often denotes associative retrieval, but in the current experiments and subsequent discussion, *context* is used to describe structured sequences of temporally associated events, such as the ABC context explained above.

Our investigation focuses on recognition memory; the ability to judge whether a stimulus has been encountered before or not. According to the dual-process model (Mandler, 1980, Montaldi and Mayes, 2010, Yonelinas, 2002), this ability can be supported by two contributing kinds of memory. Familiarity memory describes the feeling of memory that a stimulus (e.g., a face) has been encountered before, without recovering additional associative details from a previous encounter. In contrast, recollection describes the feeling of memory that is driven by the retrieval of additional non-stimulus, associative details regarding a previous encounter with a stimulus; therefore, perhaps the name of the person or the place where we met them. Despite previous assertions that the difference between familiarity and recollection reflects differences in confidence (e.g., Donaldson, 1996, Wixted and Stretch, 2004; for an extension of this view in fMRI see Squire, Wixted, & Clark, 2007), we have repeatedly shown (e.g., Kafkas et al., 2017, Kafkas and Montaldi, 2012, Montaldi and Mayes, 2010) that these two types of memory can be matched for confidence (in terms of accuracy and subjective confidence). Therefore, the critical difference between familiarity and recollection is qualitative and determined by whether recognition is accompanied by cued recall of associative information (in recollection) or not (in familiarity) irrespective of the degree of memory confidence/strength (see also Methods for instructions given to participants).

Numerous studies have revealed that familiarity and recollection can be dissociated at the behavioural level as some variables have been shown to selectively affect only one kind of memory (e.g., Brandt et al., 2006, Gardiner et al., 2006, Gardiner et al., 2001, Gardiner and Richardson-Klavehn, 2000, Norman, 2002, Rajaram, 1993). It remains unclear, however, the extent to which expectations influence familiarity and/or recollection memory, and whether any effects are common to both kinds of memory. Traditionally, dual-process models of recognition memory (Mandler, 1980, Tulving, 1985) describe recollection as strongly dependent on the context in which encoding occurs as it involves the *reinstatement* of a previous encounter with a stimulus or event, and events always occur in some kind of context. In contrast, familiarity has traditionally been referred to as an automatic (Jacoby, 1991) and context-free form of memory. Thus, it is reasonable to argue that familiarity would not be influenced by the encoding or retrieval context (see e.g., Macken, 2002). However, some evidence for familiarity memory sensitivity to background context does exist (Ecker et al., 2007, Tsivilis et al., 2001).

### 1.2 The effect of expectation on the encoding of information

The processing of information that takes place at encoding is critical for memory formation, as it may determine the extent to which successful memories are formed and the type of memory experienced later, at retrieval (Davachi and Dobbins, 2008, Kafkas and Montaldi, 2011, Paller and Wagner, 2002, Schacter et al., 1998). For example, the level of processing engaged in at encoding, determined by the nature of the task at hand when information is encoded, has been linked to different degrees of retrieval success in recognition and recall tasks (Craik and Tulving, 1975, Craik, 2002). Memory formation can also be manipulated by contextual factors that may be peripheral to the presented stimulus. For example, recognition and recall memory for word stimuli is enhanced when they are encoded in contexts congruent with pre-experimental knowledge (e.g., “Is a CORKSCREW an opener?”) than when they are described in incongruous statements (e.g., “Is SPINACH ecstatic?”) (Craik and Tulving, 1975, Schulman, 1974, Staresina et al., 2009). This *congruency effect* has been explained as a recollection enhancement effect, selective to the processing of congruent target words (Bein et al., 2015, Fisher and Craik, 1980).

Non-semantic contextual factors influencing processing at encoding that are not driven by pre-experimentally established semantic meaning should also affect memory encoding. For example, stimuli that are distinctive within a list context, perhaps due to a perceptual characteristic (e.g., larger font) or a semantic characteristic (e.g., “cat” among a list of inanimate object words) are recalled and recognised better than less distinctive items (the Von Restorff effect; Fabiani and Donchin, 1995, Rangel-Gomez and Meeter, 2013, von Restorff, 1933, Wallace, 1965). This effect suggests that the expectations that evolve during a series of temporally linked encoding episodes, may influence the on-going encoding operations and thus result in different memory outcomes at retrieval.

Indeed, in a recent study (Kafkas & Montaldi, 2015a) it was shown that encountering unexpected stimuli (as defined by the probability of occurrence of familiar and novel stimuli) at retrieval, triggered increased exploratory behaviour (revealed through eye tracking), leading subsequently to greater recollection. Moreover, this was shown to be supported, at the neural level, by increased connectivity between dopaminergic striatal/midbrain structures and the hippocampus, a structure that has a selective role in supporting recollection (e.g., Eichenbaum et al., 2007, Kafkas and Montaldi, 2012, Sauvage et al., 2008). In the same study, encountering expected stimuli resulted in enhanced subsequent familiarity-based recognition.

The differential effect that contextual expectation at encoding may have on subsequent recollection and familiarity is further explored in the current experiments (Experiments 1a and 1b). Unlike in our previous study (Kafkas & Montaldi, 2015a), the expectation status of a stimulus in the current experiments is not defined by the probability of encountering a novel or a familiar item in a recognition list – a characteristic that is also directly related to the type of decision that participants were asked to make (i.e., whether an item is old or new). Rather, in the experiments reported here, the expectation for each stimulus is based on a preceding cue, whereby the relationship between the cue and the target was either consistent (expected) or inconsistent (unexpected) with a previously learned predictive rule. Critically, this expectation manipulation was incidental to the encoding task that participants were asked to complete. Finally, in order to measure the effect of context-based expectation at encoding on later familiarity and recollection, two different encoding tasks were employed; one optimised to predominantly support familiarity (free viewing task) and the other optimised to predominantly support recollection (semantic task).

### 1.3 The effect of expectation on information retrieval

Another outstanding question is how expectations operating at retrieval may affect memory and whether this effect may be similar or different from the effect of expectations at encoding. Some theories of recognition memory regard recognition decisions as inferential or *attributional* in the sense that feelings of familiarity are mediated by an attribution derived from the perceived ease, or *fluency*, with which a stimulus is processed (Jacoby and Dallas, 1981, Jacoby and Kelley, 1987, Jacoby and Whitehouse, 1989, Westerman et al., 2002, Whittlesea and Williams, 1998, Whittlesea and Williams, 2000, Whittlesea, 1993, Whittlesea et al., 1990). Along these lines, Whittlesea (2003) proposed that recognition memory constitutes an active reconstruction of memories, based on the attribution of current experience to past events. This attribution may be modulated by characteristics of the presented stimulus, the task at hand, the context in which this process takes place, or a combination of these.

In a seminal study, Jacoby and Whitehouse (1989) explored the role played by attribution in recognition memory decisions by manipulating perceptual fluency of old and new words. They showed that fluent processing can be erroneously attributed to a previous encounter, when no other explanation for the enhanced fluency is readily available, resulting in increased old responses in a recognition task. Similar findings have been reported in other studies (e.g., Higham and Vokey, 2001, Kurilla, 2011, Olds and Westerman, 2012, Westerman, 2001, Whittlesea et al., 1990). According to the *discrepancy-attribution hypothesis*, proposed by Whittlesea and colleagues, the fluency which modulates recognition judgments, is mediated by the context in which it occurs. If the observed fluency is somewhat surprising in a given context (e.g., seeing your butcher on the bus; Mandler, 1980) and there is no other source to attribute the unexpected fluency to, then it is attributed to a previous encounter. Key to this hypothesis is the proposal that an *expectation* generated by a particular context can lead to an attribution.

Other studies (Bernstein et al., 2002, Greene, 2004, Lloyd, 2007, Lloyd et al., 2003, Westerman et al., 2002, Willems and Van der Linden, 2006, Willems et al., 2007) have reported similar results when participants’ expectations are manipulated within a recognition memory task. For example, Lloyd et al. (2003) found that a priming manipulation similar to that used by Jacoby and Whitehouse (1989) resulted in more hits and false alarms for test words encoded once rather than five times. They argued that the discrepancy between the expected fluency for the once-encoded stimuli and the experienced enhanced fluency generated by a subliminal prime probe, led to an increase in old responses for these words. Critically, however, this was not the case for words encoded five times as these were expected to be processed more fluently.

Another way to generate expectations is by manipulating the current context through the task itself, without manipulating the tested stimuli (Whittlesea and Leboe, 2003, Whittlesea and Williams, 2001, Whittlesea, 2002a, Whittlesea, 2004). In this case, the context (e.g. a sentence preceding a test word) leads to the creation of a general expectation concerning the subsequently presented stimulus. The validation of this general expectation after a small pause may lead to increased “old” responses for both old and new stimuli

In summary, attributional models (e.g., Jacoby et al., 1989, Jacoby et al., 2005) emphasise that recognition judgments may be affected by contextual variables existing at the time of retrieval. One such variable, defined by the retrieval context, as noted above, is the rememberer’s expectation, which may play a crucial role in the creation of subjective experiences of memory (Dodson and Schacter, 2002, Kelley and Jacoby, 1998, Whittlesea and Williams, 2000). Critically, it remains unexplored how expectations generated at retrieval may affect familiarity and recollection performance, and this is the focus of Experiments 2a and 2b.

### 1.4 The present study

The aim of the current set of experiments is, therefore, to directly investigate the effect of expectation on recognition memory performance, and more specifically on familiarity and recollection. This is carried out with two related pairs of experiments. Experiments 1a and 1b explored the effect the *encoding* of expected and unexpected information has on subsequent familiarity and recollection performance. Experiments 2a and 2b explored the effect that the *retrieval* of expected and unexpected information has on familiarity and recollection performance.

In both sets of experiments, a similar expectation manipulation was used with the only difference being the point at which the expectation manipulation was applied (either at encoding or at retrieval). Based on previous evidence (Kafkas & Montaldi, 2015a), we hypothesised that unexpected stimuli at encoding may lead to better recollection performance at subsequent retrieval, while the encoding of expected stimuli should not affect recollection but may have an effect on familiarity. When expectation is manipulated at retrieval an effect on recognition memory may occur (e.g., Whittlesea & Williams, 2000), but previous studies do not provide any evidence regarding the direction of the effect on familiarity and recollection. Therefore, a final aim of the current study was to explore whether expectations operating at retrieval have the same effect on memory as expectations operating at encoding. This question is critical in order to understand how expectation may influence the different stages of memory.

## 2 Methods

### 2.1 Participants

The experiments received ethical approval from the University of Manchester Research Ethics Committee. Informed consent was acquired individually for each participant and different volunteers participated in each of the experiments. Participants were assigned pseudo-randomly (alternately) to the conditions in the two experiments. A sample size of at least 27 participants per condition, as in our previous study where an expectation effect was detected (Kafkas & Montaldi, 2015a), was considered appropriate for the present experiments. Furthermore, a power analysis using *GPower* (Erdfelder, Faul, & Buchner, 1996), established that the experiments were properly powered to detect an effect [parameters: η^2^ = 0.32 (from Kafkas & Montaldi, 2015a); effect size *f =* 0.69; power = 0.99].

*Experiment 1:* A total of 56 undergraduate psychology students (6 male) with a mean age of 19.3 years (SD = 3.5) participated. Half of the participants (n = 28) were assigned to the free viewing encoding condition (Experiment 1a) and the other half (n = 28) were assigned to the semantic encoding task (Experiment 1b).

*Experiment 2:* A total of 55 participants (26 male) with a mean age of 20.65 years (SD = 2.40) participated. From this sample, 28 participants completed Experiment 2a (shallow encoding task), while 27 participants completed Experiment 2b (semantic encoding task).

### 2.2 Stimuli

**Fig. 1:**
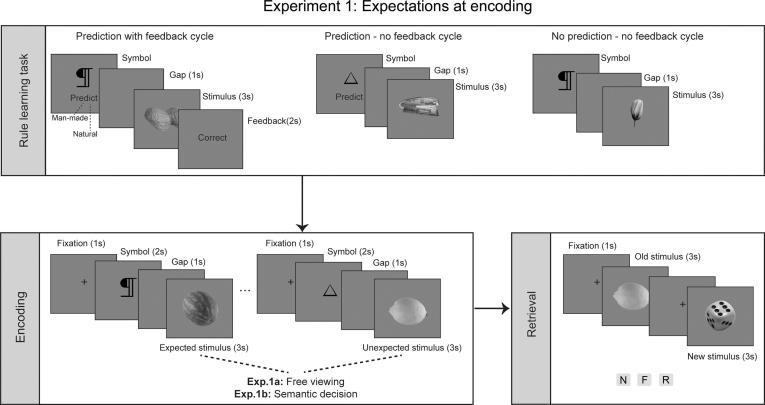
Design of Experiment 1 where expectations were manipulated at encoding. Expected and unexpected stimuli were presented at encoding and subsequent memory for these stimuli was tested at retrieval.

**Fig. 2:**
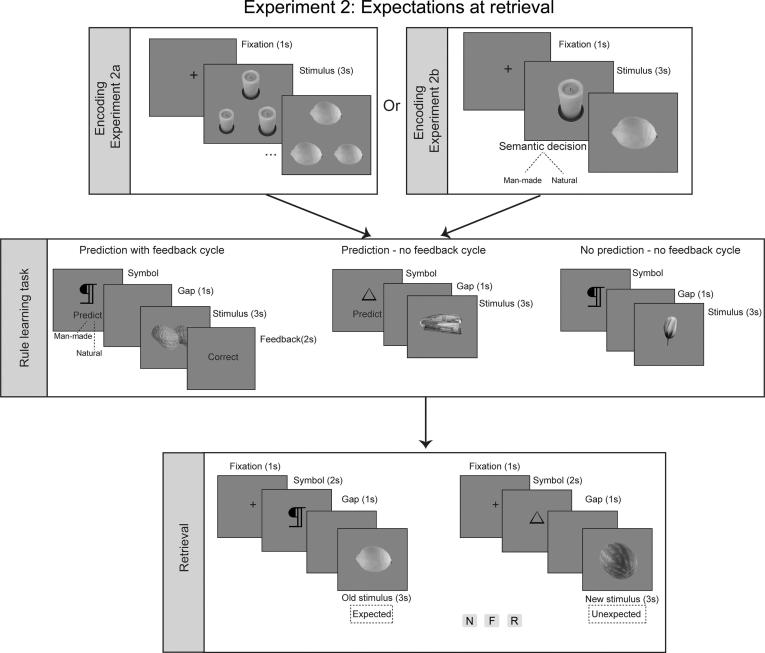
Design of Experiment 2 where expectations were manipulated at retrieval. Expected and unexpected stimuli were presented at retrieval, while participants made recognition decisions for previously studied (old) and unstudied (new) stimuli.

A set of 330 grayscale stimuli (500 × 375 pixels) depicting simple man-made and natural objects was used in this experiment. The object stimuli were collected from various online databases providing royalty-free images (e.g., the BOSS object database; Brodeur, Dionne-Dostie, Montreuil, & Lepage, 2010) and were modified accordingly so each of them fitted a uniform grey canvas measuring 500 × 375 pixels. Twelve of these stimuli were used in the practice blocks. Another 6 stimuli depicting line drawings of 6 different symbols were used for the rule-learning task and as contextual cues at encoding (Experiment 1) or at retrieval (Experiment 2; see Fig. 1, Fig. 2 and Procedure and Design below).

### 2.3 Procedure and design

The design of the different experiments reported here was very similar, with the main difference being where the expectation manipulation would occur. Specifically, expectations were generated using a rule learning task, but the manipulation of these expectations occurred either at encoding in Experiment 1 or at retrieval in Experiment 2. This meant that the sequence of the different phases was slightly different in the two experiments. In Experiment 1 (encoding manipulation), participants performed the rule learning task, then the encoding and finally the retrieval tasks. In Experiment 2 (retrieval manipulation), participants started with the encoding task, then completed the rule learning phase and finally the retrieval task. The tasks are described below for both experiments but differences are noted and are also presented in Fig. 1, Fig. 2.

**Rule Learning Task**. In order to generate expectations regarding the sequence of the events, a rule-learning task was developed. Participants learned a contingency relationship between a cued symbol (6 symbols in total) and the type of subsequent stimulus (i.e., man-made or natural). Each trial commenced with the presentation of one of the 6 symbols and participants were instructed to predict the type of stimulus (natural or man-made) that would follow the symbol. They were instructed to make a guess for the first few trials but to try to learn the rule as exposure to the cues and their stimulus type was repeated (example object stimuli were not repeated). Following the prediction cue, a man-made or a natural item (depending on the cue) appeared for 3 s. Each cue symbol was repeated (12 times in Experiment 1; 14 times in Experiment 2) across the session; each time coupled with a different stimulus of the same category (man-made/natural). That is, the stimulus type following each symbol was kept consistent within the rule-learning block for each session. The type of stimulus associated with each symbol was randomly assigned at the beginning of each session, with the requirement that 3 symbols were always followed by man-made and another 3 by natural stimuli.

The sequence of events for each trial ensured gradual learning of the correct symbol-stimulus (SS) sequence and the build-up of a general expectation for each symbol. A total of 72 SS trials were distributed across three cycles. In the first cycle (36 SS combinations with 6 symbol repetitions) each SS trial ended with a feedback screen, which informed participants about the accuracy of their initial prediction. In the next cycle, 18 SS trial combinations were presented, following the same symbol-prediction-stimulus sequence as before, but without feedback at the end. Finally, in the last 18 trials the SS trials were presented without the requirement for an explicit prediction from the participant. This last condition of the rule-learning task (without feedback and without an explicit prediction) resembled the subsequent phases of the experiments (either encoding or retrieval) and was used to smooth the transition to these tasks. In Experiment 1, participants completed the rule learning task first followed by the encoding phase, while in Experiment 2, the rule learning task was completed after the encoding phase and before retrieval. Therefore, in both cases the rule learning task was completed before the task in which expectations were manipulated.

**Experiment 1 (encoding manipulation)**. Following the rule-learning session, the participants were presented with the encoding task. Half of the participants (n = 28) completed the free viewing encoding task (Experiment 1a) and the other half a semantic encoding task (Experiment 1b). The use of two separate encoding tasks was informed by previous piloting, as well as previously published work, which established that a free viewing encoding task (as employed in Experiment 1a) resulted in recognition performance that had greater dependence on familiarity memory (see also Kafkas & Montaldi, 2011), whereas a semantic-based encoding task (as employed in Experiment 1b) resulted in recognition performance that had greater dependence on recollection memory (see also Gardiner et al., 2006, Gardiner et al., 2001, Rajaram, 1993). In both Experiment 1a and 1b the participants were told that the same symbols, as in the preceding rule-learning task, would be presented again followed by another set of natural and man-made stimuli. As with the final condition of the rule-learning task, participants were instructed that they need not make any prediction regarding the SS trials. Instead, in Experiment 1a participants were asked to simply study the stimuli carefully, while in Experiment 1b participants were instructed to make explicit man-made or natural decisions for each stimulus. Importantly, 60% of the SS combinations followed the learned rule (expected stimuli), whereas the remaining 40% of the trials violated this rule (unexpected stimuli). In both experiments, a set of 120 stimuli were presented, each for 3 s following one of the six symbols. Each new trial started with a fixation cross (1 s), followed by the symbol (2 s) then by a blank screen (1 s) and finally by the object stimulus (3 s). Each symbol was coupled with 20 different stimuli (randomly across participants) giving a total of 120 SS trials per participant at encoding.

The completion of the encoding phase was followed by two distracter tasks (one verbal and one numerical), lasting for about 15 min, and then followed by the recognition task in which a modified ‘remember/know’ procedure was used. At recognition, each participant was trained to discriminate feelings of familiarity from instances of recollection, while a response for the correct detection of foils (new stimuli) was also available to the participants. As in previous work in which familiarity and recollection responses were used at retrieval (e.g., Kafkas and Montaldi, 2012, Montaldi et al., 2006; for a review see Migo, Mayes, & Montaldi, 2012), participants were asked to report items as familiar (using a 3-point rating scale from weak to strong familiarity) when they felt that they had encountered a stimulus earlier, but the stimulus did not bring to mind any other information from the time of encoding. Accordingly, they were instructed to report a stimulus as recollected when it did bring to mind additional (non-stimulus) information associated with the encoding episode. Participants were explicitly instructed not to confuse the distinction between experiences of familiarity and experiences of recollection with the distinction between confidence of different strengths. The instructions explained that it is possible to be very confident that a stimulus has been encountered before, independent of whether it is found to be familiar or something about it is recollected (see Supplementary material for the full instructions).

A total of 120 target stimuli from the encoding phase and 60 new foils were presented at retrieval while participants were instructed to give one response [familiar (on a scale from 1 to 3 with 3 being strong familiarity), recollected, new] per stimulus within a period of 3 s. Before this recognition task, a 5-trial practice block was used to familiarise the participants with the procedure and ensure clear understanding of the instructions.

**Experiment 2 (retrieval manipulation)**. In this experiment, expectations were manipulated at retrieval. In Experiment 2a, a shallow encoding procedure was used in which participants were presented with 150 object stimuli (75 man-made and 75 natural items) and were asked in each trial to make a perceptual matching-to-sample decision. Each trial included image triplets of the same stimulus and participants were instructed to decide which of the two bottom images matched the target image presented on top. In each trial, the images were identical except that the size of one of the two bottom object pictures had been minimally modified from the top image. The assignment of the modified picture to the bottom left or the bottom right of the screen was randomly determined for each trial and participants were given 4 s to decide by using two keyboard buttons (“1″ for left and “0” for right). This task has been extensively used in previous studies and has been found to boost the frequency and accuracy of familiarity responses (e.g., Kafkas and Montaldi, 2012, Kafkas and Montaldi, 2014, Kafkas and Montaldi, 2015b, Montaldi et al., 2006). In Experiment 2b, a semantic encoding task was used in which participants were presented with the same 150 stimuli as in Experiment 2a, but in Experiment 2b they were asked to take man-made and natural decisions about the image stimuli they are seeing. In this condition, each stimulus was presented centrally for 4 s and participants used either “1” or “0” to indicate whether the depicted item was man-made or natural. The assignment of these two buttons to the two decisions was counterbalanced across participants. In both encoding conditions a centrally presented fixation cross preceded each trial for 1 s. Participants in both conditions completed a short practice block of 5 trials before starting the main encoding task.

After completing the encoding condition (either the shallow or the deep task), all participants completed the rule-learning task as described above. A retrieval (recognition) task followed, in which they were asked to report new, familiar or recollected stimuli (in relation to the encoding task). In this phase, a total of 318 stimuli consisting of 150 old (75 natural and 75 man-made) and 108 new (54 natural and 54 man-made) stimuli were presented in a random order for 3 s each, during which the recognition decision was made. Each test stimulus appeared after one of the 6 symbols that were introduced in the preceding rule-learning task. Critically, participants were explicitly instructed to focus on the recognition decision for each stimulus without giving any response to the symbol. Importantly, the SS cycle followed the rules learned in the rule learning task for only 60% of the trials (expected stimuli), whereas the remaining 40% trials violated this rule (unexpected stimuli). Each trial started with a central fixation (1 s), followed by a symbol (2 s), then a blank screen (1 s) and finally by the target stimulus (3 s).

After the end of the experiment each participant was asked to describe the aim of the experiment, and if they failed to mention anything in relation to the symbols, the predictions or expectations they were explicitly asked whether they realised the critical manipulation. Only two participants (1 in Experiment 2a; 1 in Experiment 2b and none in Experiment 1) reported noticing the violation of the symbol-stimulus rule in a subset of the test stimuli and their data were not used in the main analyses (however, inclusion of these data in an alternative analysis did not modify the main findings).

### 2.4 Data analyses

Memory performance for familiarity (collapsed across the three levels of the rating scale) and recollection responses was calculated by subtracting the false alarm rate (FA) from the corresponding hit rate for each response outcome. In Experiment 1, a mixed ANOVA with the expectation status at encoding (expected, unexpected stimuli) and memory type (familiar, recollected) as the within-subjects factors and the encoding task (free viewing, semantic task) as the between-subjects factor was conducted to compare the effect on familiarity and recollection of manipulating expectation at encoding. The data from Experiment 2 were also analysed using a mixed ANOVA with the expectation status at retrieval (expected, unexpected) and the recognition memory type (familiar, recollected) as the within-subjects factors and the encoding type (shallow or semantic) as the between-subjects factor. Post-hoc paired *t*-tests (Bonferroni-corrected) were also employed to further explore significant interactions produced by the ANOVAs. Response times (RTs) were also analysed in the same way. The same analyses were also conducted using familiarity rates calculated according to the independence assumption (Yonelinas & Jacoby, 1995) but as the results were very similar, this analysis is not reported separately. The significance level for all analyses was set at *p* < 0.05. Appropriate effect size coefficients are reported for each significant effect (*η*^2^ and Cohen’s *d* – hereafter *d*).

## 3 Results

**Fig. 3:**
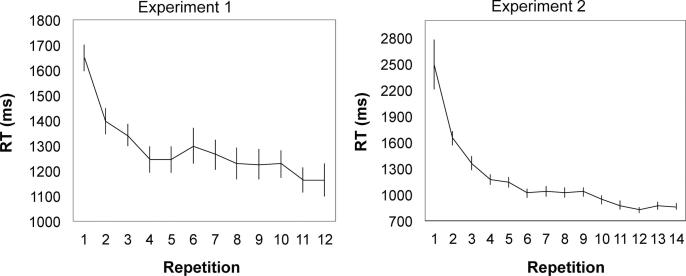
Prediction response time (RT) across the repetitions of each symbol in the rule‐learning task in Experiment 1 (encoding manipulation) and Experiment 2 (retrieval manipulation).

Rule-learning task. Participants completed the rule-learning task with a mean accuracy of 0.92 (SD = 0.08) in Experiment 1 (encoding manipulation) and of 0.91 (SD = 0.08) in Experiment 2 (retrieval manipulation), which were both significantly above chance levels of performance (Experiment 1: *t*(55) = 35.49, *p* < 0.001, *d =* 4.74; Experiment 2: *t*(54) = 36.59, *p* < 0.001, *d* = 4.93). This finding indicated effective learning of the correct SS sequences. Importantly, prediction RTs diminished significantly, across the repetitions of the symbol-stimulus sequence (Experiment 1: *F*(11, 605) = 27.00, *p* < 0.001, *η*^2^ = 0.33; Experiment 2: *F*(13, 728) = 30.77, *p* < 0.001, *η*^2^ = 0.35; Fig. 3). This significant speed-up indicates that the rule-learning task successfully created clear expectations regarding the stimulus type (man-made or natural) that would follow each symbol.

### 3.1 Experiment 1: effect of expectation at encoding on later familiarity and recollection performance

Accuracy of the semantic decision at encoding in Experiment 1b was at ceiling (M = 0.98, SD = 0.02). There was no difference in the semantic decision accuracy for expected (M = 0.98, SD = 0.03) and unexpected (M = 0.98, SD = 0.03) stimuli (*t* < 1) and no difference (*t*(27) = 1.00, *p* = 0.32) in the RTs for the two stimulus types (expected: M = 1153 ms, SD = 228 ms; unexpected: M = 1140 ms, SD = 202 ms).

The mean recognition accuracy at retrieval was 0.74 (SD = 0.09) in Experiment 1a and 0.79 (SD = 0.08) in Experiment 1b, which were both significantly above chance levels of performance (Experiment 1a: *t*(27) = 14.77, *p* < 0.001, *d* = 2.79; Experiment 1b: *t*(27) = 20.29, *p* < 0.001, *d* = 3.83). The mean proportions and RTs across the different response outcomes (hits, false alarms, misses and correct rejections) for expected and unexpected stimuli at encoding are summarised in Table 1 for both experiments.

**Table 1:** Mean proportions and response times (RTs in ms) for the different response outcomes at recognition for expected and unexpected (at encoding) stimuli in Experiment 1 (encoding manipulation).

	Proportions		RTs	
*Experiment 1a (Free viewing encoding task)*				
	Expected	Unexpected	Expected	Unexpected
Hits	0.79 (0.11)	0.75 (0.14)	1494 (236)	1464 (204)
F_Hits_	0.67 (0.14)	0.62 (0.15)	1485 (233)	1465 (218)
R_Hits_	0.12 (0.15)	0.13 (0.16)	1524 (457)	1456 (370)
FA	0.37 (0.16)		1525 (348)	
F_FA_	0.35 (0.15)		1465 (218)	
R_FA_	0.01 (0.03)		1563 (427)	
M	0.21 (0.11)	0.24 (0.14)	1416 (283)	1399 (338)
CR	0.63 (0.15)		1391 (259)	

*Experiment 1b (Semantic encoding task)*				
	Expected	Unexpected	Expected	Unexpected
Hits	0.83 (0.08)	0.86 (0.10)	1600 (158)	1602 (173)
F_Hits_	0.48 (0.19)	0.48 (0.18)	1695 (218)	1721 (248)
R_Hits_	0.35 (0.22)	0.38 (0.23)	1328 (232)	1294 (237)
FA	0.33 (0.19)		1729 (266)	
F_FA_	0.30 (0.20)		1739 (273)	
R_FA_	0.03 (0.05)		1548 (407)	
M	0.18 (0.08)	0.14 (0.09)	1569 (264)	1473 (301)
CR	0.67 (0.20)		1443 (201)	

*Note:* Numbers in the parentheses are standard deviations. F_Hits_ = familiarity hits; R_Hits_ = recollection hits; M = misses; FA = false alarms; F_FA_ = familiarity false alarms; R_FA_ = recollection false alarms; CR = correct rejections.

Memory performance (hit rate – false alarm rate) was first analysed using a mixed ANOVA with expectation status at encoding (expected, unexpected) and memory type at retrieval (familiar, recollect) as the within-subjects factors and encoding task (free viewing, semantic) as the between-subjects factor. As was expected, recollection performance was greater in the semantic decision task than in the free viewing task, whereas familiarity performance was greater in the free viewing than in the semantic encoding task, as denoted by the significant memory type by encoding task interaction (*F*(1,49) = 7.14, *p* = 0.01, *η*^2^ = 0.13). Importantly, the expectation by memory type interaction (*F*(1,49) = 6.10, *p* = 0.017, *η*^2^ = 0.11) reflected a differential effect of expectation at encoding on subsequent familiarity and recollection responses.

**Fig. 4:**
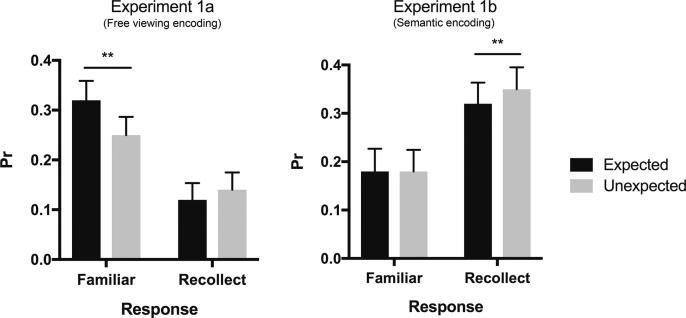
Memory performance (*Pr*: Hits – FAs) for familiarity and recollection responses for expected and unexpected stimuli at encoding in Experiments 1a (free viewing encoding) and 1b (semantic encoding). ^**^p < 0.01.

This finding was further explored by applying a series of post-hot paired *t*-tests to the two encoding tasks. As shown in Fig. 4, in the semantic encoding task, unexpected stimuli at encoding were later characterised by greater recollection performance than expected stimuli (*t*(27) = −2.78, *p* = 0.01, *d* = 0.14). On the other hand, the expectation status at encoding did not have any effect on later familiarity performance in this task (*t* < 1). However, in the free viewing encoding task expected stimuli at encoding were later characterised by greater familiarity performance than unexpected stimuli (*t*(27) = 3.04, *p =* 0.005, *d* = 0.25; Fig. 4). Memory performance for recollection responses in this task, despite being numerically higher for unexpected stimuli relative to expected stimuli, did not reach significance (*t* < 1).

The triple interaction (task × expectation × memory type) was not significant *(F* < 1) indicating that the differential effect of expectation on familiarity and recollection performance, as described above, does not statistically differ in the two encoding tasks. In other words, this finding indicates that the opposing nature of the effects of expectation at encoding on later familiarity and recollection does not simply reflect the effect of the encoding task. Instead, as shown in Table 1, the effects of expectation on memory performance, described above, stem from the differential effect of expectation on familiarity and recollection hits (F_Hits_ and R_Hits_), and consistent changes in the proportion of misses.

Finally, the analysis of RTs using a mixed ANOVA did not reveal any significant effect of expectation (*F*(1,49) = 2.17, *p* = 0.15) or significant interaction between expectation at encoding and response outcome at retrieval (*F*(1,49) = 2.97, *p* = 0.09). There was, however, a significant main effect of memory type at retrieval (*F*(1,49) = 12.45, *p* = 0.001, *η*^2^ = 0.20) and a significant interaction between memory type and task (*F*(1,49) = 15.40, *p* < 0.001, *η*^2^ = 0.24). These effects stem from the significantly faster RTs characterising recollection compared to familiarity responses in Experiment 1b (semantic task; *t*(27) = 5.67, *p* < 0.001, *d* = 1.76). In contrast, the RTs between familiarity (M = 1468 ms; SD = 232 ms) and recollection (M = 1490 ms; SD = 397 ms) responses in Experiment 1a (free viewing) did not differ significantly (*t <* 1).

### 3.2 Experiment 2: effect of expectation at retrieval on familiarity and recollection performance

In Experiment 2a, participants successfully completed the encoding (matching-to-sample) task with a mean accuracy score of 0.71 (SD = 0.12) which was significantly above chance levels of performance (*t*(27) = 8.93, *p* < 0.001, *d* = 1.69). In Experiment 2b, performance on the encoding task (man-made/natural decision) was at ceiling with a mean score of 0.96 (SD = 0.03).

The mean recognition accuracy for old and new items at retrieval was 0.72 (SD = 0.05) in Experiment 2a and 0.73 (SD = 0.08) in Experiment 2b, which were both significantly above chance levels of performance (Experiment 2a: *t*(27) = 22.40, *p* < 0.001, *d =* 4.23; Experiment 2b: *t*(26) = 15.26, *p* < 0.001, *d =* 2.94). The mean proportions and RTs for familiarity and recollection responses for expected and unexpected stimuli in Experiments 2a and 2b are summarised in Table 2.

**Table 2:** Mean proportions and response times (RTs in ms) for the different response outcomes at recognition for expected and unexpected stimuli in Experiment 2 (retrieval manipulation).

	Proportions		RTs	
*Experiment 2a (Shallow encoding task)*				
	Expected	Unexpected	Expected	Unexpected
Hits	0.84 (0.12)	0.78 (0.13)	1563 (275)	1573 (298)
F_Hits_	0.77 (0.14)	0.72 (0.15)	1546 (278)	1550 (265)
R_Hits_	0.06 (0.06)	0.06 (0.07)	1632 (427)	1670 (505)
CR	0.64 (0.17)	0.62 (0.17)	1431 (287)	1474 (282)
M	0.16 (0.09)	0.22 (0.12)	1521 (280)	1562 (322)
FA	0.36 (0.16)	0.38 (0.17)	1556 (315)	1550 (265)
F_FA_	0.35 (0.16)	0.36 (0.17)	1561 (338)	1580 (343)
R_FA_	0.01 (0.02)	0.02 (0.04)	1721 (622)	1876 (533)

*Experiment 2b (Semantic encoding task)*				
	Expected	Unexpected	Expected	Unexpected
Hits	0.80 (0.10)	0.78 (0.18)	1595 (231)	1601 (266)
F_Hits_	0.52 (0.19)	0.47 (0.20)	1669 (295)	1675 (315)
R_Hits_	0.28 (0.20)	0.31 (0.22)	1373 (262)	1372 (303)
CR	0.66 (0.23)	0.69 (0.21)	1317 (257)	1317 (253)
M	0.20 (0.10)	0.22 (0.17)	1417 (326)	1358 (297)
FA	0.34 (0.22)	0.32 (0.20)	1701 (321)	1661 (368)
F_FA_	0.31 (0.22)	0.29 (0.20)	1725 (357)	1693 (398)
R_FA_	0.03 (0.04)	0.03 (0.04)	1613 (468)	1407 (417)

*Note:* Numbers in the parentheses are standard deviations. F_Hits_ = familiarity hits; R_Hits_ = recollection hits; M = misses; FA = false alarms; F_FA_ = familiarity false alarms; R_FA_ = recollection false alarms; CR = correct rejections.

In order to explore the effect on familiarity and recollection, of the expectation status of a stimulus at retrieval, memory performance (Hits – FA rates) was analysed using a mixed ANOVA with the expectation status (expected or unexpected) and memory type (familiar or recollected) as the within-subjects factors and encoding task (shallow or semantic) as the between-subjects factor. As was expected, a significant memory type by task interaction denoted that recollection performance was greater for the items encoded in the deep encoding task than in the shallow task, whereas familiarity accuracy was greater in the shallow than the semantic encoding task (*F*(1,47) = 24.16, *p* < 0.001, *η*^2^ = 0.34). Importantly, a significant memory type by expectation status interaction was also found (*F*(1,47) = 5.44, *p* = 0.02, *η*^2^ = 0.11) denoting the differential effect of the expectation status of stimuli at retrieval on the accuracy of the familiarity and recollection responses they produced.

**Fig. 5:**
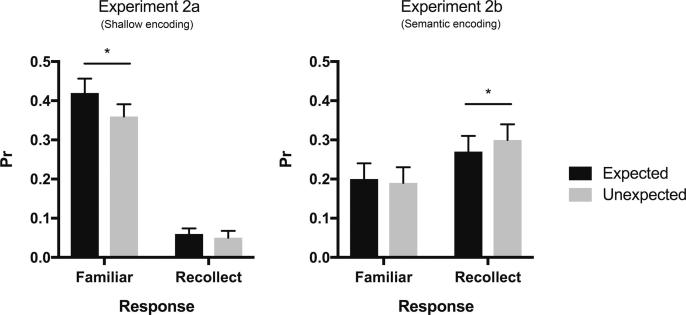
Memory performance (*Pr*: Hits – FAs) for familiarity and recollection responses for expected and unexpected stimuli at retrieval in Experiments 2a (shallow encoding) and 2b (semantic encoding). ^*^p < 0.05.

This interaction was further investigated using a series of planned contrasts within the two experiments. As shown in Fig. 5, in Experiment 2a expected stimuli were characterised by greater familiarity performance than unexpected stimuli (*t*(27) = 2.58, *p* = 0.016, *d =* 0.42), whereas recollection performance did not differ between expected and unexpended stimuli in this task (*t* < 1). In contrast, in Experiment 2b (deep task), recollection performance was higher for the unexpected stimuli at retrieval than for expected stimuli (*t*(25) = −2.72, *p* = 0.012, *d =* 0.18; Fig. 5), while familiarity performance did not differ significantly between these two stimulus types (*t* < 1). The triple interaction between expectation, memory type and task was not significant (*F* < 1) meaning that the differential effect of expectation on familiarity and recollection performance was not driven by the different encoding tasks used in Experiment 2a and 2b. As shown in Table 2, the effects of expectation on memory performance, as described above, stem from the differential effect of the expectation status on F_Hits_ and R_Hits_ and consistent changes in the proportion of misses, but critically no differences in false alarm rates.

The mixed ANOVA on RTs did not reveal any significant effect of expectation (*F* < 1) or a significant interaction between expectation status at retrieval and memory type (*F* < 1). There was only a significant interaction between memory type and encoding task (*F* = 14.82, *p <* 0.001, *η*^2^ = 0.24), indicating faster RTs for recollection responses than familiarity responses in Experiment 2b (semantic encoding; *t*(26) = 3.93, *p* = 0.001, *d =* 1.04) and a trend for faster familiarity than recollection responses in Experiment 2a (shallow encoding; *t*(24) = −1.94, *p* = 0.06, *d =* 0.41).

## 4 General discussion

### 4.1 Summary of the results

While growing evidence supports the view that expectation modulates memory, the specific form that this modulation takes is not yet known. The aim of the current experiments was therefore to explore the effect of the expectation status of both encoded and retrieved information on recognition memory, and more specifically on familiarity and recollection performance. We reasoned that this exploration may prove critical in better characterising the influence exerted by expectation on new learning and on the different kinds of memory that support retrieval. Indeed, the findings presented in this paper suggest that the level to which stimuli are expected at encoding or at retrieval has important implications for the kind of memory generated at encoding or reported at recognition. Across four experiments, we have shown that expectation has contrasting effects on familiarity and recollection performance, but interestingly, the direction of this effect was the same for expectations operating at encoding and retrieval. The findings have important implications for theories of recognition memory and for applications relating to the learning and consolidation of new information.

Experiments 1a and 1b showed that the manipulation of expectation at encoding resulted in improved recognition memory performance for expected compared to unexpected material when familiarity was the basis of recognition (Experiment 1a). In contrast, unexpected stimuli at encoding resulted in more accurate performance than expected stimuli when recollection was the basis of recognition (Experiment 1b). Consistent with this, the manipulation of expectation at test showed more accurate recognition performance for expected than unexpected stimuli when familiarity was the basis of recognition (Experiment 2a). Similarly, when recollection was the basis for recognition, unexpected stimuli at retrieval showed more accurate recognition performance than did expected stimuli (Experiment 2b). Overall, the findings show that expectations applied either at encoding or at retrieval have contrasting effects on familiarity and recollection responses, with expected stimuli selectively enhancing reliance on familiarity and unexpected stimuli selectively enhancing reliance on recollection. The mechanisms through which contextual expectation modulates memory are likely to be somewhat different for encoding and retrieval, although a degree of overlap might be predicted. These two sources of expectation effect will, therefore, be discussed separately before concluding with a tentative interpretation that draws on both.

### 4.2 Encoding expected and unexpected information

Varying the expectedness of stimuli at study appears to have a strong impact on later memory performance and recognition decisions. This probably relates to the qualitatively different type of processing that expected and unexpected stimuli receive when studied. Indeed, in a previous study (Kafkas & Montaldi, 2015a) we showed that detection of unexpected stimuli relative to expected stimuli led to increased exploratory behaviour (fixation patterns) and enhanced processing (pupil dilation) resulting subsequently in increased recollection-based recognition. This effect was characterised at the neural level by increased connectivity between striatal/midbrain structures and the hippocampus. In the current experiments, we used an expectation manipulation that is not contingent on the status of a stimulus as old or new (as in Kafkas and Montaldi (2015a)), but is instead contingent on the contextual cues preceding each trial. Enhanced learning for the unexpected stimuli was found in the present study (as in our previous study) only when recollection was the basis of recognition.

Therefore, the same mechanism as identified in the previous study (Kafkas & Montaldi, 2015a) may be in operation here; one characterised by enhanced visual exploration and dopaminergic-mediated connectivity between the hippocampus and the midbrain. This significantly extends previous findings that suggest that the more distinctive, or salient, stimuli within a list of items are remembered with greatest accuracy in recall tasks (e.g., Fabiani and Donchin, 1995, Geraci and Manzano, 2010, Johnson et al., 2006, Rajaram, 1998, Schmidt and Schmidt, 2015), by showing that this effect is also found with recognition tasks and is selective to the recollection component of recognition.

The mechanism by which distinctive information, such as the encounter of unexpected stimuli, results in greater recollection later at retrieval, may be explained using evidence from cognitive neuroscience regarding the way the hippocampus contributes to memory. Specifically, unexpected stimuli, drive the creation of highly *pattern-separated* representations at encoding (Kirwan and Stark, 2007, Norman, 2010). At retrieval, pattern separation, a hippocampal computation (LaRocque et al., 2013, Yassa and Stark, 2011), supports recollection via another hippocampal computation, pattern completion. Our findings, therefore, suggest that the encounter of unexpected stimuli boosts pattern separation at encoding, thus creating more distinctive representations of these events, resulting in greater (or more efficient) pattern completion at subsequent retrieval. Such a memory updating mechanism agrees with theoretical models stressing the interplay between prediction, novelty detection and episodic memory when recollective memories are formed (Kafkas and Montaldi, in press, Wahlheim and Zacks, in press).

Of particular novelty is the finding that alongside this unexpected-recollection effect, an expected-familiarity effect was found at encoding, whereby the subsequent recognition of expected stimuli was selectively enhanced for familiarity-based recognition but not for recollection-based recognition. Therefore, stimuli that are consistent with a learned contextually predictive rule, and are consequently expected, show a familiarity advantage at later retrieval. This effect, while suggestive of the *congruency effect,* whereby stimuli that are re-experienced in contexts congruent with previous experience are better retrieved using recognition and recall tasks (e.g., Bein et al., 2015, Craik and Tulving, 1975, Schulman, 1974, Staresina et al., 2009) is unlikely to be driven by the same mechanism. The congruency effect has been found to affect predominantly recall and recollection, rather than familiarity as found here, and also to depend on links between stimuli which have strong pre-experimental semantic associations (e.g., a “corkscrew” is a type of opener) and not new arbitrary associations as we used in the current rule-learning task. Therefore, although at first glance these effects might seem related, it is unlikely that the mechanism that underlies the congruency effect also explains our expected-familiarity effect.

Another, possibly more likely, explanation for the observed familiarity advantage for expected stimuli at encoding, may relate to the role of expectation in visual perception and its effect on enhancing the *experiential* regularity-driven similarity of the encoded stimuli. Visual discrimination and processing are enhanced when cues that predict stimuli are presented than when no cues or invalid cues are presented (e.g., Bollinger et al., 2010, Posner, 1980, Posner et al., 1980, Puri and Wojciulik, 2008, Trapp et al., 2016). In previous studies, visuospatial cues (e.g., an arrow pointing to the location of the following stimulus) or category-selective cues (e.g., the cue FACE before a face stimulus) precede a target stimulus and lead to more efficient visual processing of the stimulus that is consistent with the cue. A similar attentional mechanism may also underlie the selective familiarity enhancement observed here at encoding resulting in more efficient processing of the expected information.

The expected stimuli may, therefore, be processed more readily due to attention-mediated enhanced processing efficiency. But why this mechanism appears to benefit familiarity more than recollection remains to be explained. The answer may relate to the role of similarity, or global matching, in familiarity detection (Hintzman, 1984). In particular, it is argued that familiarity is modulated by the degree of similarity that exists between current sensory inputs and stored representations. At encoding, the fact that expected stimuli (current sensory inputs) are processed more efficiently (due to the aforementioned effect of expectation on visual perception) may result in experiencing greater similarity between sensory inputs and stored representational characteristics that are consistent with expectation. This form of *experiential* similarity thus selectively enhances later familiarity more than recollection, as the latter is driven by distinctiveness rather than similarity (as discussed above). Critically, this familiarity enhancement is likely to be more pronounced when encoding relies on the free viewing of information, as used in Experiment 1a. In contrast, a semantic encoding task (as used in 1b) would ensure that the distinctive characteristics of the unexpected stimuli were more pronounced, thus leading to the creation of pattern-separated representations and recollection.

### 4.3 The effect of expected and unexpected stimuli at retrieval

As the data clearly show, expectation not only modulated the encoding of information, but it also had an effect on recognition-based retrieval when manipulated post-encoding (Experiments 2a and 2b). In this way, manipulating the expectation status of stimuli at retrieval, just before participants made recognition decisions, again had opposing effects on familiarity and recollection. Consistent with the encoding effects, expected stimuli were more likely to be deemed familiar than were unexpected ones, whereas unexpected stimuli were more likely to be recollected than were expected stimuli. Interestingly, these effects were only observed for truly old stimuli, as reflected by increased hit rates with no change in false alarm rates. Therefore, although manipulations of contextual expectation resulted in opposing effects on familiarity and recollection performance, these effects related to the modulation of recognition for already studied materials and did not lead to alterations in the criteria set for hit detection or for false alarms. Thus, one can conclude that the mechanism, or mechanisms, underlying these effects must be acting on the processes supporting the comparison between the sensory stimulus input and the stored stimulus representation (for familiarity) and the cued (by the input stimulus) recall of the stored representation (for recollection), at the point of test, as they do not act on sensory stimulus inputs that have no previously formed internal representation (i.e., new stimuli).

As described in the Introduction, previous studies have also shown that varying the context in which recognition occurs, either in terms of the stimulus characteristics within a test list (e.g., Bodner and Lindsay, 2003, McCabe and Balota, 2007, Whittlesea and Williams, 1998) or in terms of the context that spatially or temporally surrounds a stimulus (Goldinger and Hansen, 2005, Jacoby and Whitehouse, 1989, Thavabalasingam et al., 2016, Westerman et al., 2002, Whittlesea and Williams, 2001), significantly affects recognition memory judgments. Whittlesea, 2002b, Whittlesea, 2003 has suggested that memory retrieval is the outcome of two interactive cognitive operations; a response to (or performance on) a current event, and an evaluation process which attributes the produced response to a prior occurrence in the past, taking into account prevalent contextual factors. Similarly, the source monitoring approach suggested by Johnson and colleagues (e.g., Johnson, 1997, Johnson et al., 1997, Johnson et al., 1993) places evaluation and attribution at the heart of memory retrieval. Jacoby and colleagues have also argued that attribution and inference are important components of recognition memory decisions to the extent that judgments on a past occurrence take into account characteristics of the current situation (Jacoby and Whitehouse, 1989, Jacoby et al., 1992) and in some cases exploit contextual cues to constrain the retrieval process (Jacoby et al., 2005; see also Dodson & Schacter, 2002).

Our retrieval findings are highly consistent with these arguments and further show that expectations generated within a retrieval context are subjected to evaluation and affect recognition judgments. More importantly, the manipulation of the expectation status of the tested stimuli had opposite effects on familiarity and recollection memory showing that the kind of memory experienced is contingent on contextual inferences and not on a single “strength-detection” dimension. Traditionally, theories of recognition memory have regarded familiarity as an acontextual form of memory, which responds to the absolute memory strength of a stimulus (Tulving, 1985). Instead, the manipulation in Experiment 2a shows that familiarity judgments are sensitive to contextual factors, and more specifically to the expectations formed by the contextual characteristics of the retrieval setting.

However, the source of the effect of retrieval-related expectation on familiarity and recollection memory remains to be established. This effect cannot be attributed to differential encoding of expected and unexpected information, as the expectation manipulation occurs at retrieval and therefore both categories of stimulus received the same type of encoding-related processing. It is, therefore, the retrieval context that differentially triggered the kind of memory retrieval employed when encountering expected and unexpected stimuli. For example, studied expected stimuli may have led to greater feelings of fluency than studied unexpected stimuli. This fluency may then result in the experience of greater similarity between the stored representation and the stimulus presented at retrieval, making it more likely that the stimulus be deemed familiar. In contrast, studied unexpected stimuli would not have been processed as fluently as the expected ones, albeit more fluently than new unexpected stimuli. Therefore, the encounter of old unexpected stimuli may have prompted a memory search leading to the triggering of pattern-completion processes resulting in greater recollection.

One important aspect of the design of the current experiments was that participants were instructed to focus on the recognition tasks, and at debriefing, only 2 participants reported noticing the violation of the learned symbol-stimulus sequence in a subset of the trials. This means that the effect of expectation on familiarity and recollection decisions does not necessitate conscious awareness of the operation of contextual cues but instead works implicitly. Indeed, awareness of the expectation manipulation may result in abolishing the effect on familiarity and recollection in the same way that the effect of fluency on recognition decisions is eliminated when participants can attribute it to another source, and not on past experience of the stimulus (see e.g., Jacoby & Whitehouse, 1989).

### 4.4 Conclusions and implications

Taken together, these findings suggest that expectation, defined by the encoding or retrieval context, critically affects the processes and mechanisms drawn upon to support learning and memory. The findings show that, at encoding, the level of expectation a stimulus carries ensures that the type of representation formed contains defining information that later, at test, emphasises either the similarity or the distinctiveness of a stimulus. Furthermore, the level of expectation defined by the context in which retrieval takes place informs the kind of retrieval mechanisms that are spontaneously triggered; leading either to the application of global matching and an enhanced use of familiarity-based recognition, or to rigorous pattern completion and an enhanced use of recollection-based recognition. A striking outcome of this research is that, not only is the accuracy of memory for the contextually distinctive, or unexpected events better, driven by *contextual distinctiveness enhancement mechanisms* at encoding and retrieval, but so too is the accuracy of memory for the expected events, driven instead by *contextual similarity enhancement mechanisms* at encoding and retrieval.

The finding that expected stimuli give rise to more accurate familiarity memory, whereas unexpected stimuli give rise to more accurate recollection, further highlights the discrete nature of these two kinds of memory. Experiences of familiarity and recollection respond to qualitatively different attributes of the test stimuli and their stored representations. Recollection is supported by the recovery of distinctive information, (e.g., an unexpected stimulus) which stems from the engagement of pattern separation (at encoding) and pattern completion (at retrieval) mechanisms acting selectively on an unexpected stimulus. In contrast familiarity is not simply the lack of such a recovery – and thus a weaker memory – but instead, reflects a mechanism that selectively boosts the similarity between a stored representation and the expected current sensory experience (global matching) resulting in enhanced feelings of memory.

These findings have important implications for learning protocols and learning environments, as they suggest that exposure to information that is distinctive (where distinctiveness can be defined and manipulated in a number of ways) will boost the construction and recovery of rich associative memories. On the other hand, exposure to information that follows previously learned regularities, that are readily predicted, or that can be easily accommodated into informational templates learned over time (e.g., schemas), will also have complementary benefits for memory, but these will be based on familiarity memory. Therefore, the current findings argue that learning environments and learning protocols should be designed to take advantage of both the contextual distinctiveness and the contextual similarity enhancement mechanisms, proposed here, by triggering both mechanisms optimally. More broadly, the findings also speak to previous theoretical views (e.g., Jenkins, 1979) regarding the impact of numerous factors on memory outcomes, showing that expectation could be a potentially important factor to consider in memory experiments more generally.

## Appendix A Supplementary material

Supplementary Data 2

This is open data under the CC BY license (http://creativecommons.org/licenses/by/4.0/).
