# Supplementary material for: Expectation affects learning and modulates memory experience at retrieval
Source: Cognition. 2018 Nov;180:123–34. doi: 10.1016/j.cognition.2018.07.010 (PMC6191926; doi:10.1016/j.cognition.2018.07.010)
Supplement: Supplementary Data 1 [file mmc1.docx]

**Supplementary Material**

**Article:** *Expectation affects learning and modulates memory experience at retrieval*

By Alex Kafkas & Daniela Montaldi

**Recollection and Familiarity Instructions**

Before we start the experiment, it is crucial that we carefully go through some instructions that will train you how to accurately identify the different types of memory you might experience. The two types of memory we are focussing on are *recollection* memory and *familiarity* memory, both of which may contribute to your ability to recognise a stimulus (e.g. an object) as something you have encountered previously.

**Familiarity memory**

Everyone has a pretty good idea of what it is like to find something familiar without being able to recall anything about it. For example, we have all been in the situation where we have met someone whom we recognize as familiar, although we can’t recall anything about them such as their name, where we last saw them or why we know them.

You can have the same feeling of being *sure* that you encountered something in a *specific* context (such as a study session in a testing room) although you are unable to recall anything extra about what you thought or what else happened in the room when you encountered the stimulus. Sometimes the stimulus can feel *very* familiar from the context and sometimes only *weakly* familiar.

The distinction between familiarity and recollection is very important in this experiment and it should not be confused with your feelings of *confidence*. You can be very confident that you have encountered something before, independent of whether you find it familiar or you recollect something about it.

When you recognise something as familiar you may be able to rate the strength of your memory feelings. Thus, an item may feel:

(1) Weakly familiar (e.g. in situations where you might think that something tells you that you have seen this item before or that it does not feel completely new)

(2) Moderately familiar (i.e., familiar but not quite as strong to qualify as a strong familiarity feeling) and

(3) Strongly familiar (i.e. an unquestionably familiar item you are sure you have seen it in the study session).

**Recollection memory**

Sometimes when you are shown previously studied items (e.g. objects) you will be able to recollect something specific about encountering them in the study session. In order to be recollecting you must be retrieving something that isn’t the image or the word that is in front of you or even part of it. Rather, you must be recollecting something additional to the item itself and this involves *bringing to mind* information that is not currently in front of you. The type of information you might recollect includes what you were thinking when you encountered the image or the word during the study session. For example, you might have thought that an object was particularly beautiful, and this comes back to you when you see the object again during the memory test. You might also recollect information that you might not have *explicitly* thought about during study, but also isn’t part of the stimulus. For example, you might recall that a stimulus came very early in the list or that you sneezed when the stimulus was originally presented.

However, if, when your memory is tested, you just remember seeing an item (such as an object) or even part of a stimulus in the study session, but there is no other *more specific* *extra information* that you recall about the study session, then you should say that the item is familiar (because you are recalling nothing about it that is specific to the study episode).

If you have any questions, please ask the experimenter now.

___________________________________________________________
